# Geospatial Mapping of Indoor Air Quality and Respiratory Illnesses in an Urban Slum

**DOI:** 10.7759/cureus.34890

**Published:** 2023-02-12

**Authors:** Samyak T Shah, Nayanabai Shabadi, Rohan Karkra, Vadaga V Rao

**Affiliations:** 1 Community Medicine, Jagadguru Sri Shivarathreeshwara (JSS) Medical College and Hospital, JSS Academy of Higher Education and Research (JSSAHER), Mysuru, IND; 2 General Medicine, Jagadguru Sri Shivarathreeshwara (JSS) Medical College and Hospital, JSS Academy of Higher Education and Research (JSSAHER), Mysuru, IND

**Keywords:** prana c air plus, slum, air quality index, pollution, geospatial mapping

## Abstract

Introduction

Air pollution is a well-recognized determinant of health. The general perception has focused primarily on outdoor pollution, and indoor pollution which may be due to smoking, biomass use, an extension of outdoor pollution, etc. has been neglected. It is therefore imperative to understand the levels of indoor pollution and find out if these are associated with high rates of illnesses, particularly, respiratory diseases.

Material and methods

This was a cross-sectional study involving 300 houses and 727 participants in an urban slum, selected through simple random sampling. Indoor air quality was assessed using the Prana C -Air Plus air quality monitor (Prana Air, New Delhi, India). The instrument detected formaldehyde, air quality index (AQI), temperature, humidity, PM2.5, PM10 particles, and total volatile organic (TVO) compounds. Socio-demographic details were noted, and geospatial mapping was done using Q-GIS software (www.qgis.org). A questionnaire was used to survey the residents of those houses. Ethical committee clearance was obtained before starting the project.

Results

The mean distribution of pollution parameters over the entire study area was AQI - 67.4±65.48, PM 2.5 - 37.6±35.82 μg/m^3^, formaldehyde - 0.09±0.37 mg/m^3^, PM 10 - 43.9±38.59 μg/m^3^, TVO compounds - 0.43±2.13 mg/m^3^, CO_2 _- 1128.9±323.86 ppm, temperature - 23.7±21.2 degree Celsius, and PM 1 - 24.3±20.5 μg/m^3^; 2.6% of the participants had respiratory diseases, and a significant association was found between the AQI, TVO compounds and ventilation, and respiratory diseases (p<0.05).

Conclusion

Indoor air pollution not unlike outdoor pollution can have dramatic health effects and needs to be addressed to lower the overall respiratory disease burden. The AQI, TVOC, and poor ventilation/cross-ventilation are associated with respiratory illnesses. Geospatial mapping shows a concentration of cases in areas of high pollution.

## Introduction

Clean air is a basic necessity for a healthy life. The quality of indoor air, i.e., in homes, offices, schools, government buildings, health care facilities, or any enclosed area where people spend a significant part of their life is an essential determinant of health and well-being. Hazardous pollutants produced by natural and human activities such as the combustion of fuels for cooking or heating and smoking which are contained within closed spaces lead to a broad range of health problems and may even be ultimately fatal [1]. A variety of respiratory diseases such as asthma, chronic obstructive pulmonary disorder, chronic cough, upper respiratory tract infection, pulmonary fibrosis, etc. have been linked to pollution. Other serious conditions like skin lesions, neurological manifestations, anxiety, depression, heart diseases, malignancies, cataracts, tuberculosis, pneumonia, etc. may also occur secondary to pollution, indoor or otherwise [1]. 

Indoor air pollution refers to any chemical, biological, and/or physical contamination of indoor air. Over time, experts have identified many indoor air contaminants such as nitric oxide (NO), nitrogen dioxide (NO_2_), volatile organic compounds (VOCs), sulfur dioxide (SO_2_), carbon monoxide (CO), particulate matter (PM), and biological agents such as microbes [2].

In 2010, indoor air pollution from biomass fuels alone was estimated to be responsible for nearly 3.5 million fatalities and 4.5 percent of worldwide daily-adjusted life years [3]. According to Kumar et al. in India, particulate matter <2.5 microns in diameter (PM2.5) is an important cause of respiratory illness. Increased PM2.5 levels, biomass fuel use, and the number of family members were found to be associated with increased occurrence of respiratory illness in children in their study [2].

According to Mukkannawar et al., higher particulate concentrations were observed in houses using a chulha, which involves directly burning firewood or coal for the purpose of cooking (PM2.5 1218 g/m^3^ and PM10 2993 g/m^3^), compared to a kerosene stove (PM2.5 - 416 g/m^3^ and PM10 - 491 g/m^3^) and liquid petroleum gas (LPG) stoves (PM2.5 122 g/m^3^ and PM10 341 g/m^3^), respectively [4]. Compared with the National Ambient Air Quality Standards (NAAQ), observed levels were found to be 81.8% and 97% times above for PM2.5 and PM10, respectively. People living in houses using chulhas and kerosene stoves are therefore subjected to a significantly higher risk of adverse respiratory health effects.

It is to be understood that urban health risks are distributed unequally among social groups, with most of the burden borne by certain vulnerable populations such as those living in slum areas. Therefore, the current study has been done to assess the indoor air quality of an urban slum which was followed by geospatial mapping to find out the relationship between indoor air quality and respiratory illnesses.

## Materials and methods

This was a cross-sectional study, covering 300 houses in an urban slum, as notified in government records in the Medar block. Given a 10% prevalence of self-reported respiratory symptoms amongst slum dwellers, a sample size of 139 was calculated at 95% confidence with a 5% margin of error [5]. A total of 727 people were enrolled in the study. After obtaining consent, the residents were interviewed to obtain socio-demographic details, cooking habits, personal and medical history, etc. 

The block has five sections, and houses were selected from each using the probability proportionate to size technique and simple random sampling. Indoor air quality was assessed using the Prana Air C Plus air quality monitor (Prana Air, New Delhi, India), which is a handheld portable device marketed by the Prana Air company and is a registered trademark product of theirs. The instrument, which is meant for personal use, is capable of detecting levels of formaldehyde, air quality index (AQI), temperature, humidity, PM2.5, PM10 particles, and total volatile organic (TVO) compounds. Geospatial mapping was also done using Q-GIS software (www.qgis.org). The epi-collect mobile application was used for recording latitude and longitude.

Data were entered into an excel sheet and analyzed using IBM SPSS Statistics for Windows, Version 24 (Released 2016; IBM Corp., Armonk, New York, United States) which is licensed to the institution. The descriptive data like demographic details and characteristics of households were analyzed and presented as frequencies and percentages. Inferential statistics like the independent t-test, Pearson’s correlation, and chi-square test were used to know the relationship between different air quality parameters and respiratory diseases in the study area. The statistical significance was considered at p-value < 0.05. Ethical committee clearance was obtained before starting the study.

## Results

Of the 727 participants, 51% were male and 49% were females. The majority of the participants belonged to the age group - 21-30 years (21.9%), followed by 31-40 years (16.9%); 2.6% of the subjects had respiratory diseases, 4.5% had diabetes, and 4.8% had hypertension.

The distribution of the indoor air quality parameters within the study houses and ventilation system can be seen in Table 1 and Table 2, respectively.

**Table 1:** Distribution of air quality parameters in houses (n=300) PM2.5:  Particulate matter of <2.5 microns size; PM10: particulate matter of <10 microns size; PM1: particulate matter of <1 micron size

S. No	Parameter	Mean (± Standard Deviation)
1	Air Quality Index	67.4 ± 65.48
2	PM2.5 (μg/m^3^)	37.6 ± 35.82
3	PM10 (μg/m^3^)	43.9 ± 38.59
4	Total Volatile Organic Compounds (mg/m^3^)	0.43 ± 2.13
5	CO_2 _(ppm)	1128.9 ± 323.86
6	Temperature (Celsius)	23.7 ± 21.2
7	PM 1 (μg/m^3^)	24.3 ± 20.5
8	Formaldehyde	0.09 ± 0.37

**Table 2:** Adequacy of ventilation and cross-ventilation in houses (n=300) *Fisher exact test

	Present	Absent	p Value*
Ventilation	26 (8.7%)	274 (91.3%)	0.004
Cross ventilation	24 (8%)	276 (92%)	0.005

The association between different air quality parameters and respiratory illnesses can be seen in Table 3.

**Table 3:** Association between air quality parameters and respiratory illnesses (n=300) *Independent sample t-test PM2.5: Particulate matter of <2.5 microns size; PM10: particulate matter of <10 microns size; PM1: particulate matter of <1 micron size

Parameter	Respiratory Illness		p Value*
	Present (n=19)	Absent (n=281)	
PM2.5	49.3 ± 44.54	36.9 ± 35.2	0.17
PM10	54.3 ± 45.3	43.3 ± 38.1	0.27
Total Volatile Organic Compound	2.6 ± 8.1	0.3 ± 0.99	0.001
Formaldehyde	0.2 ± 0.4	0.1 ± 0.3	0.25
Air Quality Index	100 ± 96.6	65.5 ± 63	0.03

**Figure 1:**
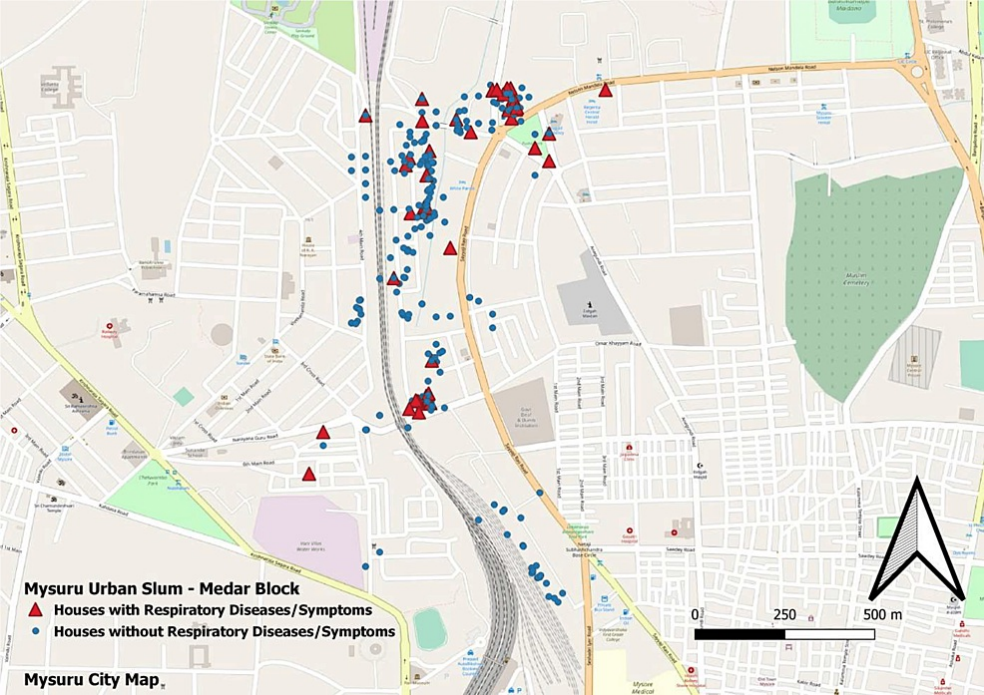
GIS mapping of study houses with and without respiratory illness/symptoms GIS: Geographic information system

**Figure 2:**
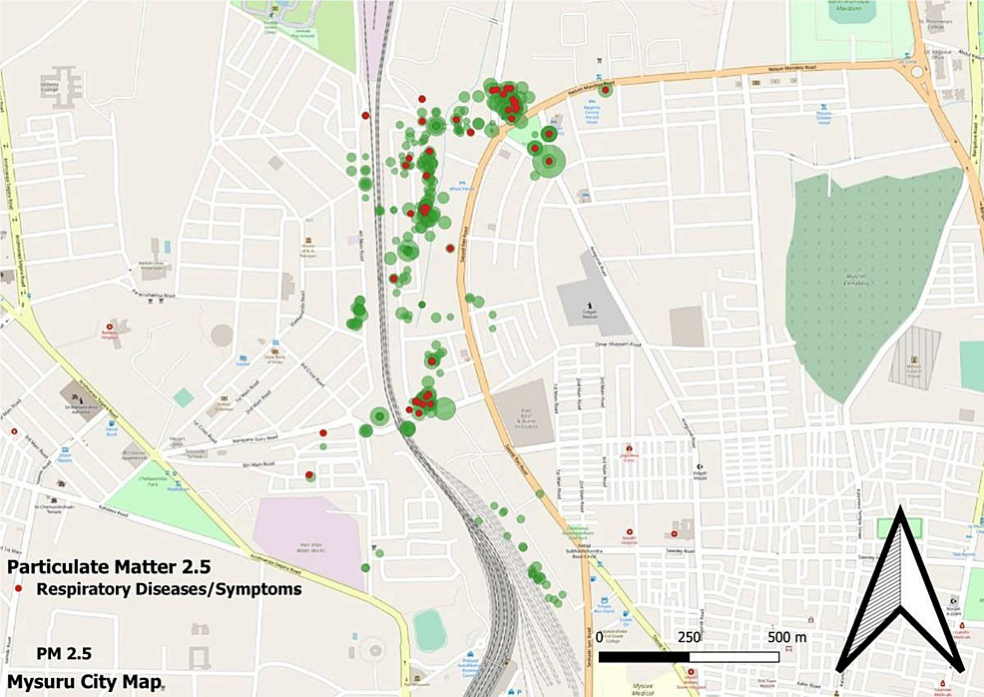
GIS mapping of study houses with respiratory illness/symptoms and levels of PM2.5 GIS: Geographic information system; PM2.5: particulate matter of <2.5 microns size

**Figure 3:**
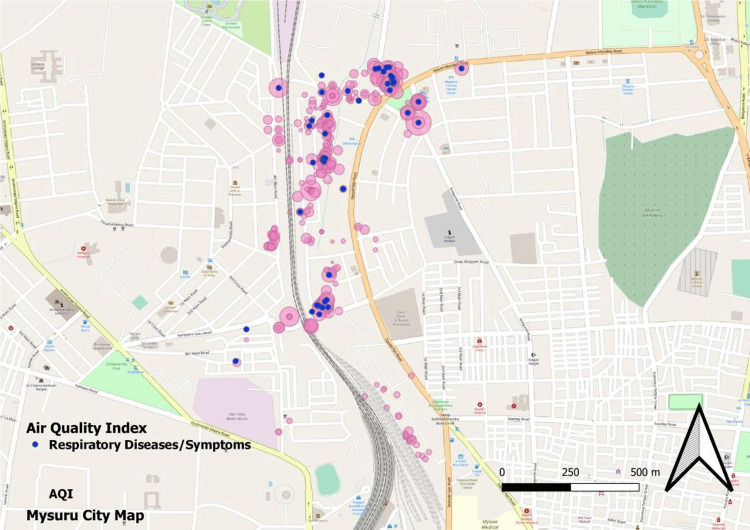
GIS mapping of study houses having respiratory illness/symptoms with the air quality index GIS: Geographic information system

**Figure 4:**
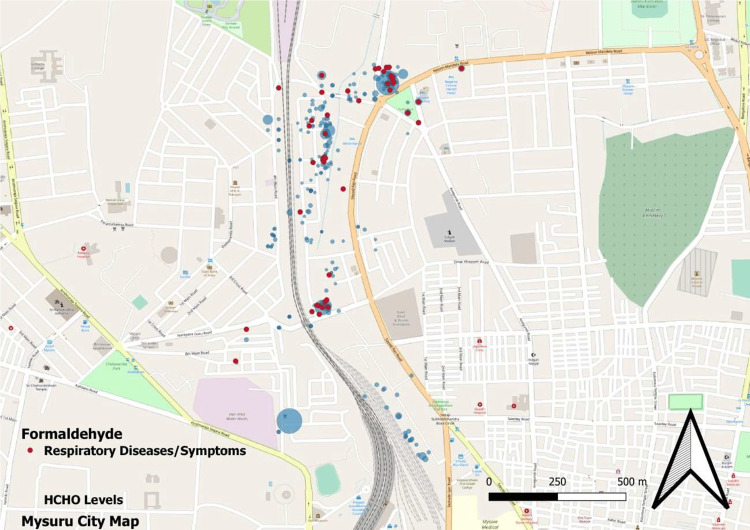
GIS mapping of study houses having respiratory illness/symptoms with formaldehyde GIS: Geographic information system

The geospatial mapping of houses is shown in Figures 1, 2, 3, and 4.

## Discussion

Indoor air pollution affects all aspects of life and people of all ages with wide-ranging multi-systemic effects on health. Every year, nearly 1.5 million lives are lost to pollution in India alone [6]. In utero exposure to household air pollutants has been shown to have dramatic health effects such as low birth weight, cognitive impairment, atopy, etc. that bear sequelae over the course of an entire lifetime [7]. Exposures to indoor air pollutants in early childhood also tend to have repercussions throughout life [8,9]. It is common sense that the respiratory system bears the maximum brunt as these pollutants get inhaled, affecting the respiratory pathway all the way from the nose to the alveoli.

The ambient air quality parameters of the city were AQI - 61, SO_2_ - 2, NO_2_ - 5, PM2.5 - 17, and PM10 - 52. The interior environment of a house is contaminated by various gases and particles produced during various human activities such as cooking, smoking, burning, candles, insecticides, incense sticks, etc [10]. The current study discovered that poor indoor air quality has a significant association with respiratory illness. Pollutants such as carbon monoxide, carbon dioxide, sulfur dioxide, nitrogen dioxide, VOCs, and other forms of hydrocarbons are released when firewood and cow dung are used as fuels [1]. In the present study, LPG was the main source for cooking at the household level but based on the sociocultural status of the family and due to economical constraints, biomass fuel was being used for the purpose of bathing. The chulhas used for cooking and boiling are present inside the house. These are not connected to smoke pipes or chimneys which in turn leads to poor indoor air quality. Hence, their residents were observed to have a variety of respiratory symptoms such as coughing and wheezing apart from comorbid conditions like diabetes, hypertension, skin allergies, and other health issues.

In our study, we found that less than 10% of the households had good ventilation and cross ventilation. This was associated with a higher risk of respiratory complaints (p<0.05). The same has also been shown in other studies [11]. A study by Park et al. also demonstrated the clear role of poor ventilation in the development of coronavirus disease 2019 (COVID-19), a respiratory illness [12]. In this study, TVO compounds and AQI were found to be significantly associated with respiratory illnesses. Studies have found that increasing AQI is associated with the development of respiratory illnesses [13]. We also attempted to do a geospatial mapping of the study area. Government bodies and scientific organizations often perform mapping of geographical areas as it helps provide a broader picture of disease and risk factor concentrations. Some studies have previously attempted geospatial mapping and found good results [14,15]. The diameter of a bubble depicted in the figures is directly proportional to the magnitude of the studied pollution parameter. It is clearly evident that the disease burden is highest in more polluted areas. Figure 2 illustrates the levels of PM2.5 (green bubbles) and the presence of respiratory symptoms (red dots). Figure 3 shows AQI (pink bubbles) and respiratory symptoms (blue dots). A concentration of cases can be seen in areas of high PM2.5 and AQI. This becomes more evident when these two figures are superimposed on Figure 1 which shows the distribution of respiratory symptoms in the studied households.

Reducing indoor air pollution exposures is a critical challenge at the confluence of home energy and public health. In practice, efficacy, cost, and the time it takes to achieve adequate intervention coverage must all be addressed before selecting an "ideal" intervention. Access to cleaner fuels has a far more significant influence on the population's health than improved stoves [16]. However, because major improvements in household access to cleaner fuels are unlikely to occur in large populations in the near future, the efficacy of ventilation and cross ventilations choices as interventions should also be considered. Identifying household air pollutants and their health implications helps us identify and correction for various health-related issues. Attempts should be made, within reasonable means, to improve indoor air quality. This would require proper ventilation and cross ventilation, avoidance of coal/cow dung chulhas, planting trees in the neighborhood and having plants in the home, limiting smoking indoors, etc. 

This study had limitations such as the limited number of households and participants, singular geographical area, COVID-19-pandemic-related restrictions, and an inability to objectively quantify disease burden, instead having to rely on history and subjective complaints alone. Pollution and associated illnesses need to be studied on a larger ecological scale and therefore our single study is itself not sufficient to make broad assertions, but instead an attempt to quantify the problem in a particular region.

## Conclusions

This study concludes that there is a significant association between poor indoor quality with respiratory illness. There is a statistically significant association between the AQI and respiratory illness (p<0.05). The association between indoor TVOC levels and respiratory illness was found to be statistically significant (p value<0.05). There was also a statistically significant association between ventilation and cross-ventilation with respiratory illness (p-value < 0.05). Geospatial mapping shows a concentration of cases in areas of high pollution.

There is a need to improve awareness among urban slum communities on the health hazards of indoor air pollution and prevent the use of solid fuels for domestic purposes.

## References

[1] World Health Organization (2010). WHO Guidelines for Indoor Air Quality: Selected Pollutants. https://www.euro.who.int/__data/assets/pdf_file/0009/128169/e94535.pdf.

[2] Kumar R, Goel N, Gupta N, Singh K, Nagar S, Mittal J (2014). Indoor air pollution and respiratory illness in children from rural India: a pilot study. Indian J Chest Dis Allied Sci.

[3] World Health Organization (2014). WHO Guidelines for Indoor Air Quality: Household Fuel Combustion. World Health Organization.

[4] Mukkannawar U, Kumar R, Ojha A (2014). Indoor air quality in rural residential area-pune case study. Int J Curr Microbiol App Sci.

[5] Brashier B, Londhe J, Madas S, Vincent V, Salvi S (2012). Prevalence of self-reported respiratory symptoms, asthma and chronic bronchitis in slum area of a rapidly developing Indian city. Open J Respir Dis.

[6] Sagar A, Balakrishnan K, Guttikunda S, Roychowdhury A, Smith KR (2016). India leads the way: a health-centered strategy for air pollution. Environ Health Perspect.

[7] Haider MR, Rahman MM, Islam F, Khan MM (2016). Association of low birthweight and indoor air pollution: biomass fuel use in Bangladesh. J Health Pollut.

[8] Christensen GM, Rowcliffe C, Chen J (2022). In-utero exposure to indoor air pollution or tobacco smoke and cognitive development in a South African birth cohort study. Sci Total Environ.

[9] Lu C, Liu Z, Liao H, Yang W, Li Q, Liu Q (2022). Effects of early life exposure to home environmental factors on childhood allergic rhinitis: modifications by outdoor air pollution and temperature. Ecotoxicol Environ Saf.

[10] Maung TZ, Bishop JE, Holt E, Turner AM, Pfrang C (2022). Indoor air pollution and the health of vulnerable groups: a systematic review focused on particulate matter (PM), volatile organic compounds (VOCs) and their effects on children and people with pre-existing lung disease. Int J Environ Res Public Health.

[11] Qian H, Zheng X (2018). Ventilation control for airborne transmission of human exhaled bio-aerosols in buildings. J Thorac Dis.

[12] Park S, Choi Y, Song D, Kim EK (2021). Natural ventilation strategy and related issues to prevent coronavirus disease 2019 (COVID-19) airborne transmission in a school building. Sci Total Environ.

[13] Kim D, Chen Z, Zhou LF, Huang SX (2018). Air pollutants and early origins of respiratory diseases. Chronic Dis Transl Med.

[14] Kumar A, Gupta I, Brandt J, Kumar R, Dikshit AK, Patil RS (2016). Air quality mapping using GIS and economic evaluation of health impact for Mumbai City, India. J Air Waste Manag Assoc.

[15] Kamińska IA, Ołdak A, Turski WA (2004). Geographical Information System (GIS) as a tool for monitoring and analysing pesticide pollution and its impact on public health. Ann Agric Environ Med.

[16] Smith KR, Frumkin H, Balakrishnan K (2013). Energy and human health. Annu Rev Public Health.

